# Evaluating the impact of a darts game intervention on cognitive function in older adults with and without mild cognitive impairment: a pilot study

**DOI:** 10.3389/fresc.2024.1327494

**Published:** 2024-02-05

**Authors:** Nami Kawabata, Tadayuki Iida, Masafumi Kunishige, Hiroshi Fukuda, Hideki Miyaguchi, Toshihide Harada

**Affiliations:** ^1^ Program in Biological System Sciences, Graduate School of Comprehensive Scientific Research, Prefectural University of Hiroshima, Hiroshima, Japan; ^2^ Department of Rehabilitation/Occupational Therapist, Faculty of Health Sciences, Hiroshima Cosmopolitan University, Hiroshima, Japan; ^3^ Department of Physical Therapy, Faculty of Health and Welfare, Prefectural University of Hiroshima, Mihara, Japan; ^4^ Department of Occupational Therapy, Faculty of Health Science Technology, Bunkyo Gakuin University, Bunkyo, Japan; ^5^ Graduate School of Information Sciences, Hiroshima City University, Hiroshima, Japan; ^6^ Department of Human Behavior Science of Occupational Therapy, Health Sciences Major, Graduate School of Biomedical & Health Sciences, Hiroshima City University, Hiroshima, Japan

**Keywords:** mild cognitive impairment, cognitive function, mental health, gravity movement, older

## Abstract

**Introduction:**

The current study investigated the relationship between the characteristics of a darts game, including the throwing motion toward a target, and mild cognitive impairment (MCI). To this end, we examined the associations between cognitive function and mental health, and the shift in center of gravity while throwing darts. In a preliminary investigation, a 1-month dart game intervention was conducted among older individuals living in the community. The participants were divided into the non-MCI and MCI groups, and the relationship between center of gravity movement during throwing and the presence of dementia was examined.

**Methods:**

The intervention lasted for 1 month and was tested on healthy older individuals (aged ≥ 65 years) recruited from the community. The Japanese version of the Montreal Cognitive Assessment and the Trail Making Test was used to assess cognitive function. Mental health was evaluated using the Kessler Psychological Distress Scale and the Subjective Well-being Inventory. The center of pressure was analyzed to determine the center of gravity shift during dart throwing.

**Results:**

The analysis of factors influencing the determination of the MCI score during the intervention revealed a tendency for the center of gravity shift to be associated as a protective factor in the non-MCI group, although this association did not reach statistical significance (odds ratio = 0.942, *p* = 0.084). In the MCI group, a significant effect of age was observed in the MCI score (odds ratio = 1.539, *p* = 0.007).

**Conclusion:**

The current findings suggest that conducting center of gravity shift testing could potentially provide a helpful tool for predicting early decline in cognitive function.

## 1 Introduction

When humans throw an object at a target, the target is recognized by the visual cortex, temporal association cortex, parietal association cortex, and prefrontal area, enabling the coordination of somatic sensations in the body and positional information regarding the target. Decisions regarding throwing are made via processing in the prefrontal area. Based on the obtained information, the motion for the most efficient throwing movement is planned in the motor association cortex, which is then carried from the primary motor area as output information via the vertebra. The result of the throwing movement is compared between the expected and actual sensory feedback in the parietal association cortex and cerebellum to identify accidental errors (1). Motor learning then determines how the next throw should be most efficiently performed for the movement task (2). The game of darts involves a repeated throwing motion directed at a target. When throwing darts, in addition to arm swinging, a forward shift of the center of pressure (COP) is necessary. Playing darts requires limb and trunk support ability, balance capacity, spatial perception ability, and center of gravity shifting. Schleien et al. (3) investigated the effectiveness of darts skill improvement and sensory feedback in disabled adults by calculating and aiming at a target. Goto et al. (4) suggested that measuring the center of gravity shifting was potentially useful for detecting cognitive decline at an early stage. Thus, the game of darts, which involves a throwing motion aimed at a target, contains elements of dual-task training and may be related to cognitive function in older people.

The 2019 guidelines from the World Health Organization recommend engaging in physical exercise to prevent cognitive decline in individuals with normal cognitive function (5). Intervention studies using dual-task training, including physical exercise, have been reported to have benefits for individuals with mild cognitive impairment (MCI) and healthy older adults (6, 7). In addition, it may be useful to evaluate not only the movement of the body during physical exercise interventions but also the process of moving the body, such as shifts in the center of gravity. In a previous study, we used a driving simulator to elucidate delayed responses in older individuals and examine the process of moving the body (8). The associations between throwing interventions, such as aiming in a darts game, and physical activity, including changes in the center of gravity, and cognitive function, are unknown.

As a pilot study, a darts game intervention study was conducted. To test the association between dementia and the movement of the center of gravity during throwing, older people living in the community were recruited, and the participants were divided into two groups: non-MCI and MCI. The participants were examined using the Japanese version of the Montreal Cognitive Assessment (MoCA-J) (9). The cohort was then divided into two groups (non-MCI and MCI) based on their MoCA-J scores. The study investigated the relationships between cognitive function and mental health characteristics, center of gravity shifting for throwing darts, and MCI score.

## 2 Methods

### 2.1 Trial design

A darts game intervention study was conducted. The participants were categorized into two groups (non-MCI and MCI) based on pre-intervention assessments. We examined the associations between MCI scores and cognitive, mental, and physical functions before and after the intervention, recognizing the pilot nature of the study. The exclusion criteria included (1) the presence of a mental disorder, such as a diagnosis of depression or dementia; (2) the inability to respond verbally to questions due to difficulties with verbal communication; and (3) the measurements needed for the study were difficult to obtain.

### 2.2 Participants

**Figure 1:**
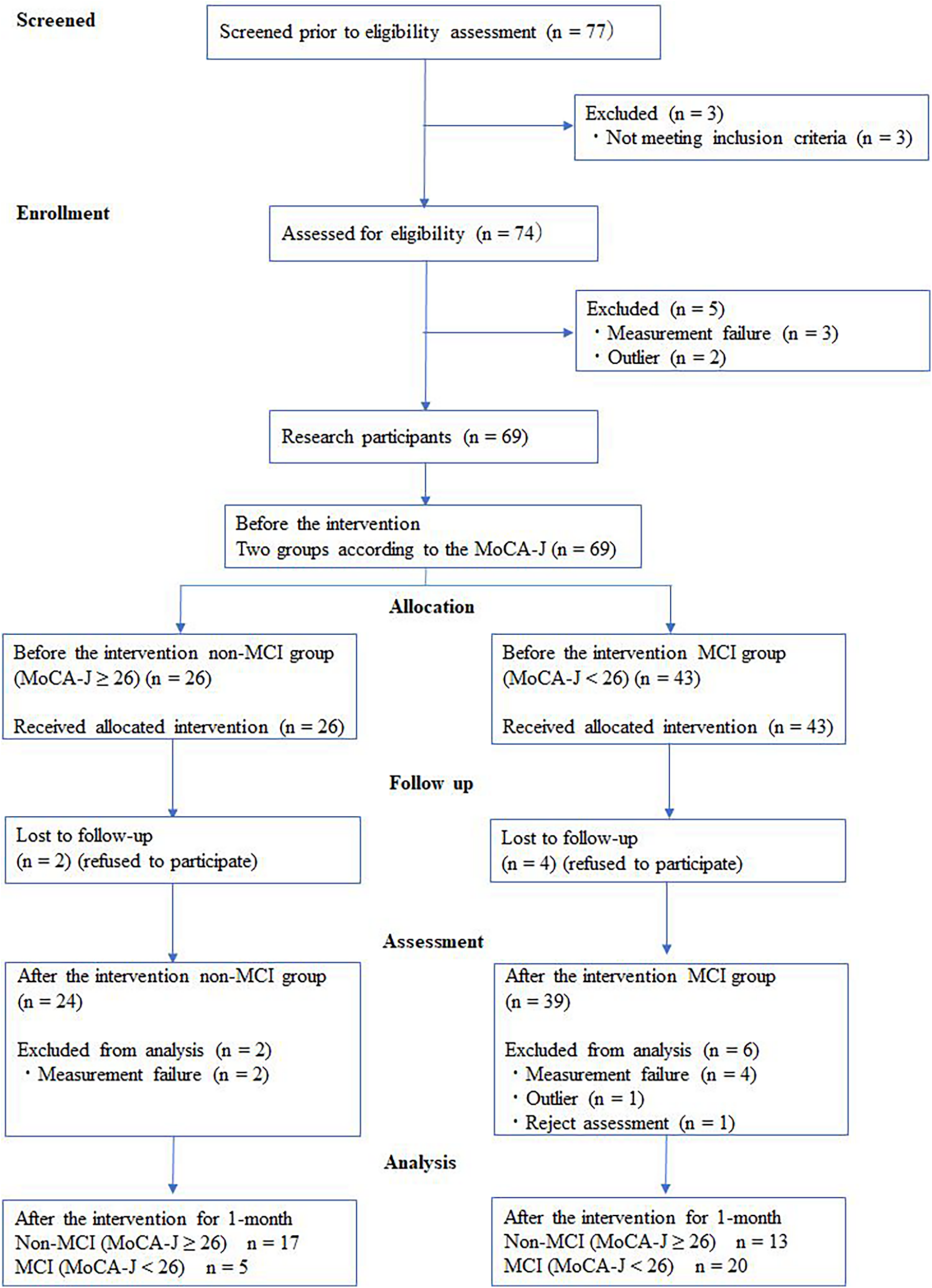
Flow diagram of participants. MCI, mild cognitive impairment; MoCA-J, Japanese version of the Montreal cognitive assessment.

The participants were healthy individuals aged 65 years or older who volunteered to participate in response to a recruitment advertisement in the M City official bulletin. This study was performed after receiving approval from the ethics committee of the Prefectural University of Hiroshima (no.: 18MH031-01), and the protocol used was in accordance with the Declaration of Helsinki. Informed consent was obtained from all individual participants included in the study. Each participant received a comprehensive explanation of the study contents and methods in advance and provided written consent. The participants were allowed to withdraw their consent at any time during the study period. Written consent was obtained from 77 individuals, 69 of whom were enrolled as participants. Six participants could not be followed up. After the intervention, we failed to obtain measurements from six participants, one participant rejected the assessment, and one participant was an outlier (Figure 1).

### 2.3 Interventions

For the darts game intervention experiment, an installation-type darts machine (DARTSLIVE2 EX, DARTSLIVE Co. Ltd., Tokyo, Japan) was installed at the community centers. The participants were given a 1-month communication program in which they played the game of darts. Using the program, darts matches were conducted with teams composed of three players, and the program was used for communication among the team members. The matches were played in rotation. Thus, the weekly activity time for each participant was approximately 1–1.5 h. The participants were expected to engage in darts match activities one to three times (non-MCI group: average = 2.71, min = 1, max = 4; MCI group: average = 2.73, min = 1, max = 4) per week. During the intervention period, the participants were instructed to continue with the same daily activities they engaged in before the intervention.

The intervention experiment activity was conducted for 29 days on average [standard deviation (SD): 2.08, min: 26, max: 32], from February to March 2019. Cognitive function and mental health, as well as physical characteristics while throwing darts, were measured before and 1 month after the start of the intervention. Teams were created to facilitate communication among the participants. The environment was adjusted to allow the participants to take part in the intervention program. The participants were divided into two groups (non-MCI and MCI) based on the results of the examinations conducted prior to the intervention. We analyzed the relationships between MCI score and cognitive function and mental health, as well as physical characteristics, before and after the intervention.

### 2.4 Outcomes

The MoCA-J and Trail Making Test were tested as cognitive functions, and the Kessler Psychological Distress Scale was measured as an assessment of mental health. The center of gravity for throwing darts and arm-swinging acceleration were measured as indicators of physical function during a game of darts.

#### 2.4.1 Cognitive function

The MoCA-J and Trail Making Test Parts A and B (TMT-A, TMT-B) were used to assess cognitive function. The MoCA-J is a tool used for measuring MCI decline. It can evaluate multiple areas related to cognitive function, such as attention function, ability to concentrate, executive function, memory, language, visuospatial ability, conceptual thinking, calculation, and orientation within a short period (approximately 10 min). The total score is 30 points. For the Japanese version, it has been reported that the normal range is 26 points or higher (9, 10). The TMT-A is used as an index for the maintenance of attention and selective ability, and the TMT-B is used for measuring executive function. Although no standard value has been set, these tasks are useful for determining effects because they employ a sequential scale for time (11, 12).

#### 2.4.2 Mental health

For the examination of depression and anxiety disorders, mental health was assessed prior to the intervention using the Japanese versions of the Kessler Psychological Distress Scale (K6) and the Subjective Well-being Inventory (SUBI). The K6 is a screening measure for mental illnesses, such as depression and anxiety disorders, and is widely used as an index for expressing the severity of various mental problems, including psychological stress, within a general investigation. The total score ranges from 0 to 24 points, with a higher total score indicating more severe mental problems (12). The SUBI is a questionnaire developed by the World Health Organization and is used to evaluate subjective well-being. The results are expressed as mental health and mental fatigue scores (health scores and fatigue scores, respectively) (13, 14).

#### 2.4.3 Physical function while throwing darts

To elucidate changes in physical function during the darts game, the center of gravity for throwing darts and arm-swinging acceleration while throwing were measured prior to starting the intervention. To determine the center of gravity shift, a kinetic balance assessment system (SS-FP40AO, SPORTS SENSING Co., Ltd., Fukuoka, Japan) was used, and the center of pressure was calculated. The direction toward the darts target was designated as direction X to acquire the trajectory length, or COPx (m). The trajectory length toward both sides of the body perpendicular to COPx was measured as COPy (m). Dart throwing was repeated three times. The average of the three throws was obtained for both COPx and COPy. For measuring acceleration, a compact wireless multifunctional sensor (TSND121, ATR-Promotions Inc., Kyoto, Japan) was used, and the average maximum acceleration (G) of three throws was calculated.

### 2.5 Sample size

The sample size was determined according to the 6-week difference in MoCA score using G-power, based on the results of a study conducted by Park et al. (15). The comparison of VAR before and after intervention in the mild dementia group, with M1 = 17.7 (mean MoCA score before intervention), SD 1 = 3.4, M2 = −20.9 (mean MoCA score after intervention), SD 2 = 3.4, two-sided *α* = 0.05, and power = 90%, indicated a sample size of 21 participants per group. This was increased to 22 (the minimum sample size of the non-MCI group) to account for potential attrition, resulting in a total sample size of 55. In this study, the participants were allowed to withdraw their consent at any time during the study period.

### 2.6 Allocation

MoCA-J scores are considered valid for MCI screening when the total score is 25 or lower (9). For the participants in the present study, those with a MoCA-J score of 26 or higher were classified into the non-MCI group, and those with a score of 25 or lower were classified into the MCI group, as MCI was suspected. MCI scores at 1 month after starting the intervention were determined in each group using the MoCA-J (Figure 1).

### 2.7 Blinding

The corresponding author created a consolidated table in which the subjects were linked, anonymized, and assigned randomized numbers. The measurer knew and measured only the randomized numbers. The occupational therapists within the research group assessed cognitive function and mental health, while the engineers in the same research group analyzed the data from dart-throwing. However, the occupational therapists and engineers who conducted the measurements did not know whether each participant was in the MCI or non-MCI group. The corresponding author, who knew whether each participant was in the MCI or non-MCI group, did not participate in conducting the survey. Although this was an intervention study of all subjects, we used a double-blind method in which neither the measurer nor the subjects knew whether they were in the MCI or non-MCI group.

### 2.8 Statistical methods

Participants' physical characteristics, cognitive function measurements, mental health investigation results, physical function measurements while throwing darts before the intervention, and the average MoCA-J scores (SD) after the intervention were obtained. Student's *t*-tests were used to compare the average scores before the intervention between the non-MCI and MCI groups. In each group, the effects of risk factors were examined with binominal logistic regression analysis using the MCI score at 1 month after starting the intervention as the objective variable (dependent variable). The explanatory variables for physical characteristics included age, stature, and body mass prior to the intervention. For cognitive function measurements, the time taken to complete the TMT-A and TMT-B prior to the intervention was used as an explanatory variable. For the mental health investigation, K6 scores prior to the intervention and SUBI health/fatigue scores were used as explanatory variables. Regarding the physical function measurements while throwing, COPx, COPy, and acceleration prior to the intervention were used as explanatory variables. In the analyses of cognitive function, mental health, and physical function while throwing, data were adjusted for age before the intervention. The SPSS software package, ver. 25.0 J (IBM Japan, Ltd., Tokyo, Japan), was used for all analyses and statistical processing of measured data. Regarding the level of significance, values of 0.05 or lower were considered to indicate statistical significance.

## 3 Results

### 3.1 Verification of data reliability and validity

All study participants exhibited no physical limitations that affected the implementation of the darts game. Of the 77 participants, 22 participants obtained a predicted failure on the screening test, refused to participate, or were lost to follow-up and were therefore excluded from the analysis.

### 3.2 Comparisons of physical characteristics and measurements of cognitive function and mental health with physical function while throwing prior to the intervention between the non-MCI and MCI groups

Significant differences between the two groups were observed, with significantly lower age (73.5 ± 4.6 vs. 79.3 ± 5.2 years, *p* < 0.01), completion time of TMT-B (91.0 ± 37.0 vs. 150.7 ± 59.4 s, *p* < 0.01), and SUBI health score (39.1 ± 5.7 vs. 41.2 ± 7.0 points, *p* < 0.05) in the non-MCI group compared with those in the MCI group. COPx was significantly higher in the non-MCI group (0.19 ± 0.05 vs. 0.16 ± 0.05 m, *p* < 0.01) compared with that in the MCI group. No significant differences were observed for the other items (Table 1).

**Table 1:** Comparisons of physical characteristics and measurements of cognitive function and mental health with physical function while throwing before intervention between the non-MCI and MCI groups.

	non-MCI group (*n* = 22)		Mean ± SD	MCI group (*n* = 33)	*p*-value
	Mean ± SD	Min–Max		Min–Max	
Physical characteristics					
Age (years)	73.5 ± 4.6	65.0–83.0	79.3 ± 5.2	69.0–89.0	<.001
Stature (m)	1.56 ± 0.07	1.43–1.72	1.54 ± 0.06	1.42–1.68	0.425
Body mass (kg)	55.19 ± 9.4	36.0–83.2	54.2 ± 7.2	43.0–72.0	0.675
Cognitive function					
MoCA-J (points) before intervention	27.2 ± 1.2	26.0–30.0	22.2 ± 3.0	14.0–25.0	<.001
After 1-month intervention	25.6 ± 2.1	21.0–30.0	22.33.3	15.0–27.0	<.001
TMT-A (seconds)	40.3 ± 14.2	19.0–74.0	51.0 ± 25.5	26.0–126.0	0.055
TMT-B (seconds)	91.0 ± 37.0	51.0–224.0	150.7 ± 59.4	71.0–300.0	<.001
Mental health					
K6 (points)	3.3 ± 3.4	0.0–11.0	3.8 ± 4.0	0.0–15.0	0.943
SUBI: health score (points)	39.1 ± 5.7	28.0–51.0	41.2 ± 7.0	24.0–55.0	0.034
SUBI: fatigue score (points)	51.6 ± 5.6	39.0–62.0	51.3 ± 6.0	38.0–62.0	0.221
Physical function					
COPx (m)	0.185 ± 0.045	0.102–0.299	0.157 ± 0.052	0.078–0.259	0.031
COPy (m)	0.091 ± 0.034	0.033–0.164	0.092 ± 0.039	0.039–0.171	0.890
Acceleration (G)	7.015 ± 2.059	3.325–11.000	6.969 ± 2.209	3.226–10.697	0.938

SD, standard deviation; Min, minimum; Max, maximum; MoCA-J, Japanese version of the Montreal cognitive assessment; TMT-A/B, the trail making test part A and part B; K6, Japanese version of the Kessler psychological distress scale; SUBI, the subjective well-being inventory; COPx, the orbital length toward the darts target; COPy, the orbital length toward both sides of the body.

The *p*-values were determined using Student's *t*-test.

### 3.3 Results of binominal logistic regression analysis using MCI score obtained 1 month after starting the intervention as the objective variable (dependent variable) in both groups

Table 2 shows the relationships between physical characteristics and measurements of cognitive, mental, and physical function while throwing before the intervention. It also presents the MCI scores 1 month after starting the intervention in the non-MCI group. The analysis of factors influencing the determination of the MCI score during the intervention revealed a tendency for COPx to be associated as a protective factor in the non-MCI group, although this association did not reach statistical significance (odds ratio = 0.942, *p* = 0.084).

**Table 2:** The results of logistic analysis of the MCI scores after 1 month of intervention in the non-MCI group.

	Odds ratio	95% CI	*p*-value
Physical characteristics			
Age (years)	0.983	0.766–1.262	0.889
Stature (m)	0.000	0.000–1.45 × 10^4^	0.224
Body mass (kg)	1.043	0.905–1.202	0.562
Cognitive function^a^			
TMT-A (seconds)	0.923	0.817–1.042	0.195
TMT-B (seconds)	1.008	0.985–1.031	0.521
Mental health^a^			
K6 (points)	1.090	0.836–1.420	0.526
SUBI: health score (points)	1.077	0.847–1.370	0.544
SUBI: fatigue score (points)	0.898	0.696–1.157	0.404
Physical function^a^			
COPx (m)	0.942	0.881–1.008	0.084
COPy (m)	1.034	0.967–1.106	0.328
Acceleration (G)	2.432	0.646–9.146	0.189

CI, confidence interval.

^a^ Adjusted for age.

Table 3 shows the results of the logistic analysis of the association between the presence of MCI and physical characteristics, measurements of cognitive, mental, and physical function after 1 month of intervention in the MCI group. Age was found to be significant (odds ratio = 1.539, *p* = 0.007).

**Table 3:** The results of logistic analysis of the MCI scores after 1 month of intervention in the MCI group.

	Odds ratio	95% CI	*p*-value
Physical characteristics			
Age (years)	1.553	1.130–2.134	0.007
Stature (m)	0.955	0.000–3.07 × 10^7^	0.996
Body mass (kg)	1.036	0.877–1.224	0.678
Cognitive function^a^			
TMT-A (seconds)	0.973	0.919–1.030	0.347
TMT-B (seconds)	1.021	0.994–1.049	0.126
Mental health^a^			
K6 (points)	0.904	0.687–1.190	0.473
SUBI: health score (points)	1.109	0.954–1.290	0.178
SUBI: fatigue score (points)	0.946	0.808–1.107	0.486
Physical function^a^			
COPx (m)	0.980	0.954–1.006	0.123
COPy (m)	1.004	0.974–1.035	0.775
Acceleration (G)	1.661	0.867–3.182	0.126

CI, confidence interval.

^a^ Adjusted for age.

## 4 Discussion

In the present study, an interventional experiment using a darts game was performed for 1 month with community-dwelling older people. The participants were divided into a non-MCI group and an MCI group based on MoCA-J results obtained before starting the intervention. The prevalence rate of MCI has been reported to be approximately 50% among community-dwelling older people in Japan (16). Among the 77 participants in the study, 43 had MCI. Therefore, the number of participants in the MCI group in this study was considered to be appropriate. We investigated the associations of cognitive, mental health, and physical function characteristics determined prior to the intervention with MCI scores after the intervention had finished. The results suggested that the present data were reliable (i.e., the prevalence rate of MCI among the elderly was 43 out of 77, or 56%). All participants received adequate training in throwing darts before the experiment started, which may have also contributed to the validity of the data.

Logistic analysis of the MCI scores 1 month after starting the intervention revealed a tendency for the center of gravity shift while throwing toward the target to be associated as a protective factor in the non-MCI group, although this association did not reach statistical significance (COPx: odds ratio = 0.942, *p* = 0.084). To successfully play darts, a sense of the distance between the target and the player as well as a perception of space are required. When the target is recognized, visual information is carried from the occipital lobe to the upper parietal lobule in the parietal association cortex and is then integrated with somatic sensation and converted to information regarding the target position for body coordination. That information is then carried to the dorsal premotor cortex, where the most efficient movement for throwing the dart is planned. Simultaneously, visual information is carried from the occipital lobe to the lower parietal lobule of the parietal association cortex and the ventral premotor cortex, where the most suitable hand-grip pattern for the throwing is selected and planned. This motion planning is projected from the primary motor area to the vertebra as output information. An efference copy of the reach-grip motion for this situation is converted to prediction information for movement results, to compare between predicted and actual sensory feedback in the parietal association cortex and cerebellum, and to extract any errors (1). As a result, exercise-based learning on how to efficiently perform the motor task of dart throwing occurs (2). Because movement speed is decreased in older people (17), not only arm swinging but also a forward shift of the center of gravity is needed for aiming at a distant target. With advancing age and diminishing cognitive capabilities, studies have consistently reported a decline in coordination between the hands and eyes, resulting in a deterioration of motor performance coupled with increased variability (8, 18). Furthermore, a correlation was reported between general cognitive efficiency and spatial mental transformation skills, and its influence has been observed in cognitive tasks associated with driving (19). In addition, previous research has highlighted the dynamic evolution of motor processes throughout an individual's lifespan. Thus, it may be useful to quantify the process of movement using metrics such as COPx. Considering these factors, we speculate that the non-MCI participants learned the motion involved in repeating a throwing movement three times by making use of physical function and spatial perception before the intervention started, even without any prior experience throwing darts, allowing them to plan the motion and actually throw the darts while shifting their center of gravity in the direction of the target. However, the potential influence of interest and familiarity in relation to the game of darts cannot be excluded. Such an influence may reduce the association with the center of gravity shifting. In future studies, examining participants during a darts game with a longer period of intervention will be needed.

The present results for the non-MCI group indicated that the center of gravity shift toward the target was likely to be a protective factor against cognitive functional decline. Furthermore, the center of gravity shift toward the target while throwing noted before the start of the intervention was greater in the non-MCI group compared with that in the MCI group. This notion is in accord with previous findings showing that center of gravity shift testing is a valuable tool useful for identifying cognitive function decline at an early stage (4). A shortened walking stride, slowed walking speed, and decline in dynamic balance ability have been noted in older individuals with MCI (20–22). In addition, a study of body image in older people suggested that the discrepancy with reality was affected by aging (23). The association between the center of gravity shift during dart throwing and cognitive function shown in the present study suggests the importance of maintaining physical function before MCI onset, particularly through continuous physical activity and exercise.

TMT-B completion time was significantly shorter in the non-MCI group compared with that in the MCI group. The TMT has been widely used for assessing attention function, and past studies have found that the time for completion of both the TMT-A and TMT-B increases with age (24, 25). This finding is in accord with the previous studies mentioned above, indicating that the attention function of the non-MCI group was better than that of the MCI group. Furthermore, SUBI health scores were significantly lower in the non-MCI group compared with those in the MCI group. The SUBI health score is widely used to evaluate the positive effects of the subjective sense of well-being, with higher scores indicating a more positive sense of well-being (13, 14). A previous study reported that subjective well-being tends to increase at the age of approximately 60 years and is then maintained until very old age (26). The lower SUBI health scores in the present non-MCI group are in accord with this previous finding, as the participants were younger compared with those in the MCI group.

In a logistic analysis of MCI scores at 1 month after starting the intervention, age was confirmed to be a significant risk factor for cognitive function decline in the MCI group. Furthermore, the age of the MCI group was significantly higher than that of the non-MCI group. Regarding dementia, an increased prevalence is associated with aging, with some differences based on region. In Japan, approximately 10%–15% of individuals in their early 80s, 15%–25% in their late 80s, and 30% or more of those aged 90 or older are estimated to be affected by dementia (27). Thus, aging is the greatest risk factor for developing dementia. Similar findings were obtained for the MCI group in the present study, and improvement became more difficult as the age at the time of MCI onset increased. In addition, the primary prophylaxis to prevent developing MCI itself might be more important than the secondary or tertiary prevention after onset. The present results support the importance of preventing cognitive function decline at a younger age while individuals are still healthy.

### 4.1 Limitations

This study involved several limitations that should be considered. First, the participant cohort comprised older people from a single city. Thus, it may be inappropriate to generalize the findings to the whole population, although the participants' stature and body mass were similar to the average values reported in a recent national survey conducted in Japan (28). In addition, the presence of only 17 (30.9%) male participants suggests a potential bias in the male-to-female ratio. Sex differences have been reported for the diagnosis of amnestic MCI (29), which may limit the generalizability of the present results. The intervention period in this study was only 1 month, and the effectiveness of the intervention for improving cognitive function is questionable. In the study conducted by Manera et al. (30), participants showed interest in the game during the 1-month validation in the MCI group. We expect that the effect can be evaluated in this study as well, although for a short period of 1 month. However, the potential effects of interest and habituation in relation to the darts game cannot be excluded. Therefore, the association with the center of gravity shift may have become smaller. In future studies, verification of the current findings using a darts game with a longer period of intervention may be useful.

## 5 Conclusion

A 1-month intervention study using a darts game as an exercise intervention was conducted with older people living in the community. We examined the associations between cognitive function measurements, mental health, and center of gravity shift while throwing darts before and after the intervention. The participants were divided into the non-MCI and MCI groups, based on the MoCA-J scores prior to the intervention. The results in the non-MCI group indicated that the center of gravity shift while throwing toward the target was a potential protective factor, and this finding was marginally statistically significant. In the MCI group, no significant associations were found between cognitive, mental, and physical function, except for age. These findings suggest that the results of the center of gravity shift test may be indicative of early cognitive function decline, highlighting the importance of early primary prophylaxis to prevent the onset of MCI.

## Data Availability

The original contributions presented in the study are included in the article/Supplementary Material; further inquiries can be directed to the corresponding author.
